# Celebrating a decade of sound science: *Physiological Reports*

**DOI:** 10.14814/phy2.15903

**Published:** 2024-01-01

**Authors:** Kim E. Barrett, Damian M. Bailey

**Affiliations:** ^1^ University of California Davis School of Medicine Sacramento California USA; ^2^ University of South Wales Cardiff Wales UK

It is a pleasure for us as Editors‐in‐Chief of The Physiological Society sister journals to offer some comments in celebration of the 10th anniversary of the publication of *Physiological Reports*. One of us (KEB) was a member of the Publications Committee of the American Physiological Society (APS) when the concept for the journal was conceived. On hearing that The Physiological Society (TPS) had similar ideas, a few high‐level meetings quickly convinced all parties that the fortunes of the fledgling publication could only be enhanced if we joined forces as equal partners. The development of a fully open access journal to which content could be cascaded from the societies' other titles, as well as accepting de novo submissions, was a relatively novel strategy at the time. But the rapid pace of change in scholarly publishing, which is appearing to increase exponentially, has brought many additional similar efforts on the part of Society and commercial publishers alike. There has also been an explosion in biomedical titles generally, the rise of open access mega‐publishers such as MDPI and Frontiers (with marketing tactics aimed at both authors and reviewers that sometimes feel algorithmic and irritating, if not predatory), an increased acceptance of preprints as preliminary (or sometimes the only) tangible products of research projects, and intriguing experiments in peer review and other editorial processes. Moreover, society journals are facing some strong headwinds and financial risks as funders increasingly mandate that work be made immediately and freely available to all upon publication. TPS and APS are clearly not immune to these market forces. Nevertheless, *Physiological Reports* is positioned to ride the wave of changes in the industry, both by virtue of its well‐established Gold Open Access publishing model, but also as a result of its links to two distinguished founding scholarly societies and subsequent relationships for manuscript transfers that have been brokered with other societies or other physiology titles produced by its publisher, Wiley.

From the beginning, moreover, *Physiological Reports* has carved out an important identity that allows it to play a unique role for the physiological community. Not only does it publish typical mechanistic reports of the types submitted to the journals we lead (*The Journal of Physiology* and *Experimental Physiology*), but it proudly and actively solicits reports of studies that in the words of Editor‐in‐Chief, Jo Adams (Adams, 2022) can be “descriptive, correlative, confirmatory, or overlapping with prior publications.” Indeed, *Physiological Reports* even permits the publication of well‐conducted but ultimately “negative” studies. The journal accordingly is a major tool to battle the so‐called “replication crisis” that has beset biomedical science in general, and physiology in particular. Thus, in combatting a system (both within journals and in our institutions and funding bodies) characterized by biases that tend to favor publication of positive results, *Physiological Reports* helps to provide a more complete and accurate state of the field. It can be argued that, in the end, it is collective rather than individual evidence that provides us with true insights. Similarly, publication of pre‐trial registrations (e.g., Registered Report category in *Experimental Physiology*) that can be followed with the results in *Physiological Reports*, even if the findings are negative, permits us to assess the field more systematically, forming a stronger and more reliable basis for physiological “metascience.” Indeed, as stated by the Editor‐in‐Chief herself, Physiological Reports is a journal “standing openly for sound science” (Adams, 2022).

*Physiological Reports* exceeded the expectations of APS, TPS and even its publisher from its inception. It has done an extraordinary job in delivering an author‐centered experience, delivering rapid and frequently positive editorial decisions on manuscripts referred from its partner titles, especially if the manuscripts are accompanied by their original reviews following transfer. Those partner titles now include not only our TPS‐published journals and the research journals of APS, but also others, as noted above. The editors of *Physiological Reports* can swiftly inform authors which are the points, if any, from the reviews that should be addressed to secure acceptance, often without a need for additional experimentation. In addition, *Physiological Reports* has an enviable reputation for handling de novo submissions efficiently and humanely. Simply put, the journal is a crucial lynchpin of a publishing ecosystem that comprehensively serves the physiological community.

We are proud to stand behind *Physiological Reports* as equal partners in this ecosystem, with each of our journals serving its own particular purpose and for the collective good of our discipline. We encourage authors of manuscripts given a “reject and refer” decision at our Journals to consider directly transferring to *Physiological Reports*. Most importantly, all of our journals are edited by working scientists **for** working scientists, not only encompassing the members of our societies, but physiological scientists worldwide. The strength in numbers represented by this ecosystem is certainly a hedge against the financial realities of a rapidly emerging system where all journals likely will need to flip to a Gold Open Access model that rests on Article Publication Charges (APC) rather than subscription revenues—indeed, *Experimental Physiology* has already made this transition with new strategies helping it navigate the path ahead notwithstanding ongoing transformative agreements. This federation should also allow us to remain viable in the face of rising competition as well as rapid consolidation among commercial publishers, making our partnership with *Physiological Reports* more vital than ever while increasing the reach of our collective scientific output. We look forward to growing this collaboration across TPS' flagship journals for many additional decades to come (Figure 1).

**FIGURE 1:**
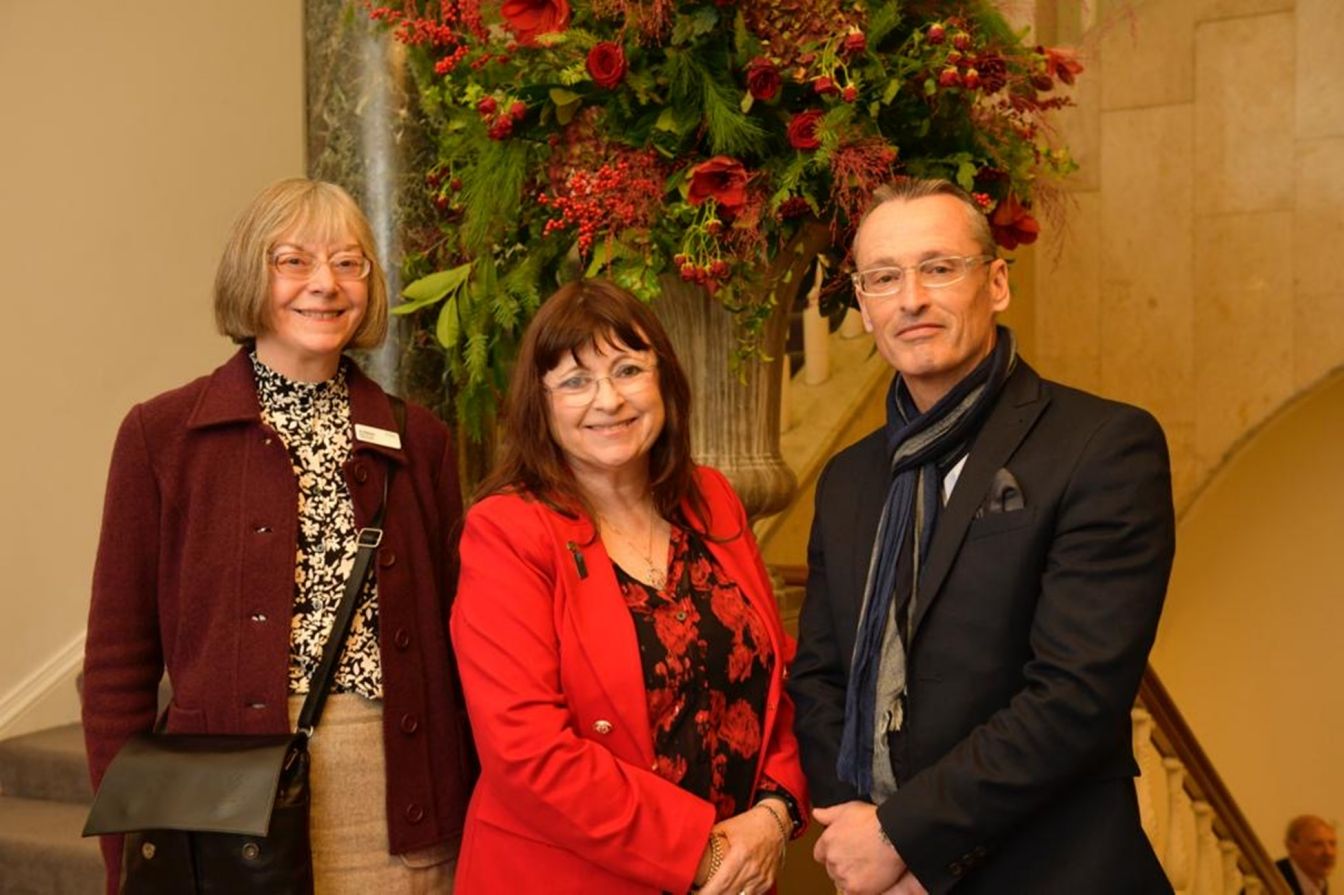
The authors meeting in London with Dr. Jo Adams (left), Editor‐in‐Chief of *Physiological Reports*, in December 2023 to discuss collaborative initiatives. Photograph courtsey of Mr. Alexander Orrow.

## FUNDING INFORMATION

DMB is supported by a Royal Society Wolfson Research Fellowship (#WM170007).
